# Sofosbuvir Based Regimens in the Treatment of Chronic Hepatitis C with Compensated Liver Cirrhosis in Community Care Setting

**DOI:** 10.1155/2018/4136253

**Published:** 2018-08-01

**Authors:** Vijay Gayam, Amrendra Kumar Mandal, Mazin Khalid, Osama Mukhtar, Arshpal Gill, Pavani Garlapati, Mowyad Khalid, Mohammed Mansour

**Affiliations:** ^1^Department of Medicine and Gastroenterology, Interfaith Medical Center, 1545 Atlantic Avenue, Brooklyn, NY 11213, USA; ^2^Department of Medicine, Wayne State University/Detroit Medical Center, Detroit, Michigan, USA

## Abstract

**Background:**

Direct-acting antiviral (DAA) drugs have been highly effective in the treatment of chronic hepatitis C (CHC) infection. We aim to evaluate the treatment response of Sofosbuvir based DAA in CHC patients with compensated liver cirrhosis as limited data exists in the real-world community setting.

**Methods:**

All the CHC patients with compensated liver cirrhosis treated with Sofosbuvir based DAAs between January 2014 and December 2017 in a community clinic setting were retrospectively analyzed. Pretreatment baseline patient characteristics, treatment efficacy with the sustained virologic response at 12 weeks posttreatment (SVR12), and adverse reactions were assessed.

**Results:**

One hundred and twelve patients with CHC infection and concurrent compensated cirrhosis were included in the study. Black patients represented the majority of the study population (64%). Eighty-seven patients were treated with Ledipasvir/Sofosbuvir (LDV/SOF) ±Ribavirin and 25 patients were treated with Sofosbuvir/Velpatasvir (SOF/VEL). Overall, SVR 12 after treatment was achieved in 90% in patients who received one of the two DAA regimens (89.7% in LDV/SOF group and 92% in SOF/VEL group). SVR 12 did not vary based on age, sex, body mass index, baseline HCV viral load, HCV/HIV coinfection, type of genotype, and prior treatment status. Apart from a low platelet count, there were no other factors associated with a statistical difference in SVR 12(*p=*0.002) between the two regimens. Fatigue (35%) was the most common adverse effect and no patients discontinued treatment due to adverse effects.

**Conclusion:**

In the community care setting, Sofosbuvir based DAAs are safe, effective with high overall SVR, and well tolerated in patients with CHC patients with compensated liver cirrhosis.

## 1. Introduction

Anywhere between 3.2 and 5 million people in the United States have a chronic hepatitis C (CHC) infection which if untreated can develop into cirrhosis, hepatocellular carcinoma, and death [1, 2]. CHC is the most common indication for a liver transplantation and a more common cause of death than all other notifiable infectious etiologies combined in the United States [3, 4].

Successful treatment of CHC, also described as a sustained virological response (SVR), is defined as an absence of detectable HCV RNA 12 weeks after the completion of treatment. CHC patients who achieve SVR have both lower rates of complications and lower overall mortality [5]. Until recently, CHC treatment was primarily based on interferon-based regimens, but disappointing response rates, particularly amongst patients with advanced liver disease, necessitated the need for a new regimen [6].

Newer drugs that directly inhibit the virus replication cycle have led to the advent of oral HCV treatment regimens known as direct-acting antiviral (DAAs) [7]. In existing clinical trials, DAA regimens have demonstrated cures rates over 90% of patients who have chronic CHC infection and concurrent cirrhosis.

Due to the relative novelty of DAA regimens, there is a paucity of literature establishing safety, tolerability, and efficacy of DAA in the real-world community care setting. As a result, we aim to analyze DAA's safety, tolerability, and efficacy in an inner-city community care setting with an emphasis on the most commonly used DAA in patients with cirrhosis.

## 2. Materials and Methods

The study protocol was approved by the Institutional Review Board (IRB) and the patients were recruited from a community hospital: Interfaith Medical Center

### 2.1. Patients

The 112 consecutive patients with CHC treated between January 2015 and December 2017 were included in this retrospective cohort study and received at least twelve weeks of treatment with one of the recommended combination regimens in standard doses for chronic HCV infection. Two different treatment regimens were used in our study, Ledipasvir (LDV) 90 mg/day+ Sofosbuvir (SOF) 400 mg/day ±Ribavirin (RBV) 1000 mg/day if <75kg and 1200 mg/day if ≥75kg, and Sofosbuvir (SOF) 400 mg/day+ Velpatasvir (VEL) 100 mg/day. Duration of treatment ranged from 12 weeks (n=98) to 24 weeks (n=14) depending on prior treatment status and existing cirrhosis.

### 2.2. Study Assessments

Treatment safety and tolerability were assessed by reviewing documented adverse events, treatment completion rates, reduction in the dosage, and discontinuation of medications. Laboratory studies were conducted both pretreatment and posttreatment. Laboratory values were then compared to look for any abnormalities associated with antiviral therapy. A diagnosis of liver cirrhosis was based on the amalgamation of clinical symptoms, laboratory parameters including FibroSure score ≥ 0.75, imaging modalities (ultrasonography and or computed tomography scan), and histopathology when indicated. We included compensated cirrhosis, defined as the absence of ascites, jaundice, hepatic encephalopathy and variceal bleeding according to the American Association for the Study of Liver Diseases, in this study.

Treatment response was assessed with HCV RNA viral load (IU/ mL) at each of 4 weeks after initiation of treatment, completion of treatment, and 12 weeks after completion of treatment. Viral load was assessed using COBAS® AmpliPrep/COBAS® TaqMan® HCV Quantitative Test, v2.0 (Roche molecular diagnostics) with the lower limit of quantification (LLOQ) of HCV RNA 15 IU/ml. SVR 12 was defined as an undetectable viral load 12 weeks after the completion of treatment.

### 2.3. Statistical Analysis

The SPSS® statistics software package (IBM SPSS® statistics version 21, USA) was used for statistical analysis. Values were expressed as mean ± SD and mean quantitative values were analyzed using Student's t-test. Differences in qualitative values were analyzed by Chi-square test. All* p* values were two-tailed and* p-*value < 0.05 was considered significant. One-way analysis of variance (ANOVA) was used to determine whether there were differences among the group means. Multivariable logistic regression was performed only in variables with a* p-*value < 0.05 in univariate analysis.

## 3. Results

### 3.1. Baseline Characteristics

Baseline characteristics are shown in Table 1. Mean age of the patients in the study was 60.7 years with age, ranging from 28 to 82 years. The majority of the patients were males 67 (75%), black 71(63.4%), and treatment-naive (76.8%). Genotype 1 was predominant, consisting of over 89.3% of the study population. Nine (23.2%) patients had received prior treatment. Thirty-five patients (31.3%) had a history of diabetes; 51 (45.5%) had hypertension; 12 (10.7%) patients had coronary artery disease, 24 patients had HCV/HIV coinfection. None of the patients had a hepatocellular carcinoma, previous liver transplant, or decompensated cirrhosis.

### 3.2. Treatment Regimens

**Figure 1:**
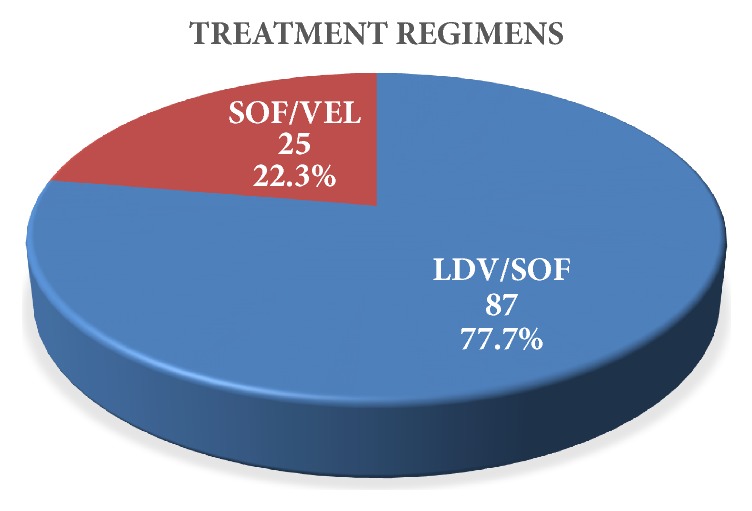
Treatment regimens.

Among the 112 patients with CHC infection, 87(77.7%) patients were treated with Ledipasvir/Sofosbuvir, and 25 (22.3%) were treated with Sofosbuvir/Velpatasvir (Figure 1).

### 3.3. Overall Virologic Response to Treatments

**Figure 2:**
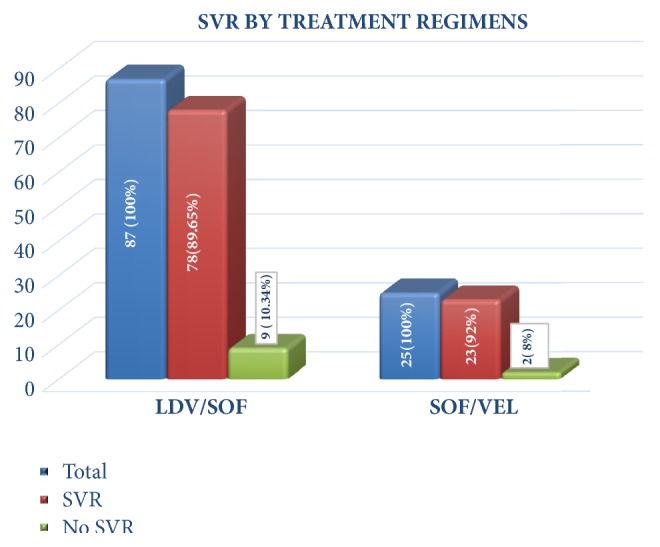
Treatment response in both groups by overall SVR 12.

The overall sustained virological response (SVR) on completion of treatment was 90 %. SVR 12 in the two treatment groups is depicted in Figure 2. In the univariate analysis, there were characteristics noted in the patients who achieved SVR12 as compared to those who did not achieve SVR. Higher mean BMI, higher Child-Pugh score, low mean platelet count, low mean albumin, and low mean bilirubin level were all more likely to be seen in patients who achieved SVR 12 in the univariate analysis. After adjusting baseline characteristics in multivariable logistic regression models, only low platelet count was found as a significant predictor of treatment response (*p*-value =0.020). Interestingly, SVR was not affected by HCV RNA levels or previous treatment status (Table 2).

### 3.4. Virologic Response in LDV/SOF Group

In this group, 78(89.7%) achieved SVR 12. HCV viral load and type of HCV genotype did not have any impact on overall SVR. Additional comorbidities including diabetes mellitus, chronic kidney disease, and HIV seropositivity did not impact SVR rates as shown in Table 3.

### 3.5. Virologic Response in SOF/VEL Group

In this treatment group, 23 (92%) achieved SVR (Table 4). Although this value is encouraging, it may not be truly significant due to a small sample size. HCV viral load and type of HCV genotype did not have any impact on overall SVR. Additional comorbidities including diabetes mellitus, chronic kidney disease, and HIV seropositivity did not impact SVR rates as shown in Table 4.

### 3.6. Safety

Only a small percentage of patients developed minor side effects from DAA treatment, and none required discontinuation of therapy. Fatigue, headache, rash, and thrombocytopenia were the most common adverse events observed. Anemia was seen only in patients treated with LDV/SOF + Ribavirin combination. There were no serious adverse events seen amongst all regimens. There was no statistically significant difference of adverse events noted between the two treatment groups. A complete list of adverse events is shown in Table 5.

## 4. Discussion

Antiviral therapy has rapidly evolved for the treatment of chronic HCV infection. The primary endpoint is to achieve SVR 12, which in turn diminishes the risk of decompensation, hepatocellular carcinoma, and death.

DAAs offer the most effective regimens for the majority of HCV infected patients. Selection of regimens is primarily based on the genotype, cirrhosis status, and other various individual patient factors. The efficacy of DAAs for patients with compensated cirrhosis is now well validated [8–10]. We studied the two most commonly used regimens, LDV/SOF and SOF/VEL.

We noted an SVR of 89.7% in LDV/SOF group which is comparable to ION-2 trial where SVR of 86% was reported in patients with cirrhosis [8]. Another trial (SIRIUS trial) reported a higher SVR of 97% (HCV genotype 1) in compensated cirrhosis. This discrepancy may be explained by the difference in duration of therapy; the SIRIUS trial patients each received 24 weeks of therapy versus our study in which patients received only 12 weeks of therapy [11].

Our second regimen was SOF/VEL which is currently approved for the treatment of all genotypes (as per AASLD guidelines). In the present study, 23 patients (92%) achieved SVR in SOF/VEL which is similar to the study by Asselah et al. They reported SVR in 96% of patients with HCV related cirrhosis after 12 weeks of treatment [12]. Our study also showed SVR rates consistent with ASTRAL-3 and ASTRAL-1 trial. These trials also used SOF/VEL regimen in a similar group of patients with overall SVR rate of 88% and 98%, respectively [12, 13]. Our study also exhibited similar response rates to the recent POLARIS-3 trial in which cirrhotic patients were given SOF/VEL for 12 weeks. They demonstrated an excellent SVR of 96 % in HCV genotype 3 infections [14].

Thrombocytopenia was associated with a lower SVR (*p*=0.02) in our study. Several studies have documented that chronic HCV infection is strongly associated with thrombocytopenia. Between 64% and 76% of patients with fibrosis and cirrhosis related to chronic HCV infections exhibited thrombocytopenia, as compared to only 6% in noncirrhotic patients [15, 16]. Thrombocytopenia is strongly related to the degree of liver fibrosis owing to low thrombopoietin levels and splenic sequestration of blood cells as a direct result of elevated portal pressure, especially among patients with advance fibrosis or cirrhosis [17–19]. Thrombocytopenia has also been associated with a higher risk for cirrhosis-related morbidity and mortality [20, 21]. Thus, early treatment of HCV infection before the development of thrombocytopenia may improve overall treatment outcomes.

Our study was supported by Suwantarat et al. who reported that thrombocytopenia was associated with lower SVR (*P*=0.05) [22]. In contrast to our study, they used interferon-based therapy and greater cytopenia was hypothesized to be a marker for greater TNF activity, which translated into a significant SVR variation. Importantly, several studies including Coverdale et al. and Taniguchi et al. reported that improvement in platelet count correlated with the regression of hepatic fibrosis following SVR 12 among patients with chronic HCV infection [23, 24].

The present study had a high HIV/HCV coinfection rate of 21.4%, but there was still a high overall SVR. This is consistent with the literature, as Osinusi et al. also demonstrated an overall high SVR (97%) in patients with HIV/HCV coinfection [25].

There were no major adverse effects in our study leading to a discontinuation of therapy, which was also noted in the ION-1 trial in which no patient discontinued LDV/SOF. Common adverse events in the present study were fatigue, headache, insomnia, and nausea. ASTRAL-1, 2, and 3 with SOF/VEL also had similar adverse effects [12, 13].

We noted an excellent sustained virological response rate in patients with compensated cirrhosis irrespective of genotypes in both treatment groups, and these results are consistent with the landmark literature described above. Our study is distinct from most studies in current literature as it establishes real-world effectiveness, tolerability, and safety of Sofosbuvir based regimens in CHC patients with compensated cirrhosis. This is in contrast to the literature, which still relies heavily on clinical trials. Limitations of the study are the small sample size and retrospective nature.

## 5. Conclusion

In the community care setting, Sofosbuvir based DAAs remain a safe, effective, and well tolerated in patients with chronic CHC patients with compensated liver cirrhosis.

## Figures and Tables

**Table 1:** Demographic and clinical characteristics of patients at baseline by treatment regimen.

**Characteristics**	**All patients** **(*n* = 112)**	**Treatment Regimens**		***p-*value**
		**LDV/SOF**	**SOF/VEL**	
		**(*n* = 87)**	**(*n* = 25)**	
**Sex**				0.629
Male	67 (59.8)	51 (58.6)	16 (64.0)	
Female	45 (40.2)	36 (41.4)	9 (36.0)	

**Age** (years)	60.7 (28-82)	61.6 (28-82)	57.2 (34-73)	0.076

**Age group** (Years)				0.234
< 65	74 (66.1)	55 (63.2)	19 (76.0)	
≥ 65	38 (33.9)	32 (36.8)	6 (24.0)	

**BMI** (Kg/m^2^)	29.0 (18.5-47.0)	28.7 (18.5-47.0)	30.2 (20-43)	0.242

**BMI** (Kg/m^2^)				0.488
< 30	65 (58.0)	52 (59.8)	13 (52.0)	
≥ 30	47 (42.0)	35 (40.2)	12 (48.0)	

**HCV Genotype**				0.131
1a	66 (58.9)	52 (59.8	14 (56.0)	
1b	34 (30.4)	28 (32.2)	6 (24.0)	
2	5 (4.5)	4 (4.6)	1 (4.0)	
3	3 (2.7)	2 (2.3)	1 (4.0)	
4	4 (3.6)	1 (1.1)	3 (12.0)	

**HCV RNA** (IU/mL)				0.330
< 800,000	31 (27.7)	26 (29.9)	5 (20.0)	
≥ 800,000	81 (72.3)	61 (70.1)	20 (80.0)	

**Prior treatment**				0.086
Naïve	86 (76.8)	70 (80.5)	16 (64.0)	
Experienced	26 (23.2)	17 (19.5)	9 (36.0)	

**Comorbidities**				
Diabetes	35 (31.3)	25 (28.7)	10 (40.0)	0.284
Hypertension	51 (45.5)	37 (42.5)	14 (56.0)	0.233
Coronary artery disease	12 (10.7)	9 (10.3)	3 (12.0)	0.728
Kidney disease	9 (8.0)	8 (9.2)	1 (4.0)	0.681
Chronic anemia	5 (4.5)	2 (2.3)	3 (12.0)	0.073
HIV seropositive	24 (21.4)	21 (24.1)	3 (12.0)	0.192

**MELD score**				0.195
< 10	62 (55.4)	51 (58.6)	11 (44.0)	
≥ 10	50 (44.6)	36 (41.4)	14 (56.0)	

**Laboratory tests**				
Hemoglobin (g/dL)	13.4 (9.2-17.5)	13.5 (9.2-17.5)	13.0 (10.0-16.0)	0.278
Platelets (x1000/mL)	140.7 (23-316)	143.4 (43-316)	131.5 (23-216)	0.371
Albumin (g/dL)	3.5 (1.3-4.7)	3.5 (1.3-4.7)	3.5 (2.2-4.5)	0.899
AST (IU/L)	82.3 (16-210)	83.3 (16-210)	78.9 (20-179)	0.639
ALT (IU/L)	77.8 (12-264)	79.5 (12-264)	72.1 (13-200)	0.510
Bilirubin (mg/dL)	1.2 (0.3-4.9)	1.2 (0.3-4.9)	1.1 (0.5-3.2)	0.752

Data are presented as mean (range) or number (percentage).

BMI, body mass index; HCV, hepatitis C virus; RNA, ribonucleic acid; APRI, AST-to-platelet ratio index; MELD, model for end-stage liver disease; AST, aspartate transaminase; and ALT, alanine transaminase.

**Table 2:** Demographic and clinical characteristics of patients at baseline by treatment response.

**Characteristics**	**All patients** **(*N* =112)**	**Treatment Response**		**Univariate Analysis** ***p-*value**	**Multivariate Analysis** ***p-*value**
		**SVR**	**No SVR**		
		**(*n* =101)**	**(*n* =11)**		
**Age** (years)	60.7 (28-82)	60.7 (28-82)	60.0 (45-67)	0.797	NA

**Age group**				0.747	NA
< 65	74 (66.1)	66 (65.3)	8 (72.7)		
≥ 65	38 (33.9)	35 (34.7)	3 (27.3)		

**Sex**				0.194	NA
Male	67 (59.8)	58 (57.4)	9 (81.8)		
Female	45 (40.2)	43 (42.6)	2 (18.2)		

**BMI** (Kg/m^2^)	29.0 (18.5-47.0)	29.4 (19.0-47.0)	25.8 (18.5-32.5)	0.050^*∗*^	0.085

**BMI** (Kg/m^2^)				0.353	NA
< 30	65 (58.0)	57 (56.4)	8 (72.7)		
≥ 30	47 (42.0)	44 (43.6)	3 (27.3)		

**HCV Genotype**				0.150	NA
1a	66 (58.9)	57 (56.4)	9 (81.8)		
1b	34 (30.4)	34 (33.7)	0		
2	5 (4.5)	4 (4.0)	1 (9.1)		
3	3 (2.7)	3 (3.0)	0		
4	4 (3.6)	3 (3.0)	1 (9.1)		

**HCV RNA** (IU/mL)				1.000	NA
< 800,000	31 (27.7)	28 (27.7)	3 (27.3)		
≥ 800,000	81 (72.3)	73 (72.3)	8 (72.7)		

**Prior treatment**				1.000	NA
Naïve	86 (76.8)	77 (76.2)	9 (81.8)		
Experienced	26 (23.2)	24 (23.8)	2 (18.2)		

**Comorbidities**					
Diabetes	35 (31.3)	31 (30.7)	4 (36.4)	0.738	NA
Hypertension	51 (45.5)	46 (45.5)	5 (45.5)	0.995	NA
Coronary artery disease	12 (10.7)	12 (11.9)	0	0.604	NA
Kidney disease	9 (8.0)	8 (7.9)	1 (9.1)	1.000	NA
Chronic anemia	5 (4.5)	4 (4.0)	1 (9.1)	0.410	NA
HIV Seropositive	24 (21.4)	20 (19.8)	4 (36.4)	0.245	NA

**MELD score**				0.060	NA
< 10	62 (55.4)	59 (58.4)	3 (27.3)		
≥ 10	50 (44.6)	42 (41.6)	8 (72.7)		

**Laboratory tests**					
Hemoglobin (g/dL)	13.4 (9.2-17.5)	13.3 (9.2-17.5)	13.9 (11.6-15.9)	0.276	NA
Platelets (x1000/mL)	140.7 (23-316)	146.4 (23-316)	88.6 (43-177)	0.002^*∗*^	0.020
Albumin (g/dL)	3.5 (1.3-4.7)	3.6 (1.3-4.7)	3.2 (2.2-4.1)	0.042^*∗*^	0.873
AST (IU/L)	82.3 (16-210)	81.6 (16-198)	88.7 (42-210)	0.585	NA
ALT (IU/L)	77.8 (12-264)	77.2 (12-204)	83.9 (27-264)	0.666	NA
Bilirubin (mg/dL)	1.2 (0.3-4.9)	1.1 (0.3-3.9)	1.6 (0.8-4.9)	0.043^*∗*^	0.821

Data are presented as mean (range) or number (percentage).

*∗*Only variables with the *p-*value < 0.05 in univariate analysis were assessed.

BMI, body mass index; HCV, hepatitis C virus; RNA, ribonucleic acid; APRI, AST-to-platelet ratio index; MELD, model for end-stage liver disease; AST, aspartate transaminase; and ALT, alanine transaminase.

**Table 3:** SVR 12 rates in patients receiving LDV/SOF by population subgroup.

**Response**	**SVR 12 Rate**	**Univariate Analysis** ***p*-value**	**Multivariate Analysis** ***p*-value**
**Overall**	78/87 (89.7)		

**Age group**		1.000	NA
< 65	49/55 (89.1)		
≥ 65	29/32 (90.6)		

**Sex**		1.000	NA
Male	44/51 (86.3)		
Female	34/36 (94.4)		

**BMI** (Kg/m^2^)		0.304	NA
< 30	45/52 (86.5)		
≥ 30	33/35 (94.3)		

**HCV Genotype**		0.009^*∗*^	0.983
1a	45/52 (86.5)		
1b	28/28 (100)		
2	3/4 (75.0)		
3	2/2 (100)		
4	0/1 (0)		

**HCV RNA** (IU/mL)		1.000	NA
< 800,000	23/26 (88.5)		
≥ 800,000	55/61 (90.2)		

**Prior treatment**		0.682	NA
Naïve	62/70 (88.6)		
Experienced	16/17 (94.1)		

**Comorbidities**			
Diabetes	21/25 (84.0)	0.272	NA
Hypertension	33/37 (89.2)	1.000	NA
CAD	9/9 (100)	0.589	NA
Kidney disease	7/8 (87.5)	1.000	NA
Chronic anemia	2/2 (100)	1.000	NA
HIV Seropositive	18/21 (85.7)	0.681	NA

**MELD Score**		0.154	NA
< 10	48/51 (94.1)		
≥ 10	30/36 (83.3)		

**ALT** (IU/L)		0.678	NA
< 40	17/18 (94.4)		
≥ 40	61/69 (88.4)		

Data presented as number/total number (percent).

*∗*Only variables with the *p-*value < 0.05 in univariate analysis were assessed.

BMI, body mass index; HCV, hepatitis C virus; RNA, ribonucleic acid; APRI, AST-to-platelet ratio index; MELD, model for end-stage liver disease; and ALT, alanine transaminase

**Table 4:** SVR 12 rates in patients receiving SOF/VEL by population subgroup.

**Response**	**SVR 12 Rate**	**Univariate Analysis** ***p*-value**	**Multivariate Analysis** ***p*-value**
**Overall**	23/25 (92.0)		

**Age group**		1.000	NA
< 65	17/19 (89.5)		
≥ 65	6/6 (100)		

**Sex**		0.520	NA
Male	14/16 (87.5)		
Female	9/9 (100)		

**BMI** (Kg/m^2^)		1.000	NA
< 30	12/13 (92.3)		
≥ 30	11/12 (91.7)		

**HCV Genotype**		0.789	NA
1a	12/14 (85.7)		
1b	6/6 (100)		
2	1/1 (100)		
3	1/1 (100)		
4	3/3 (100)		

**HCV RNA** (IU/mL)		1.000	NA
< 800,000	5/5 (100)		
≥ 800,000	18/20 (90.0)		

**Prior treatment**		1.000	NA
Naïve	15/16 (93.8)		
Experienced	8/9 (88.9)		

**Comorbidities**			
Diabetes	10/10 (100)	0.500	NA
Hypertension	13/14 (92.9)	1.000	NA
CAD	3/3 (100)	1.000	NA
Kidney disease	1/1 (100)	1.000	NA
Chronic anemia	2/3 (66.7)	0.230	NA
HIV Seropositive	2/3 (66.7)	0.230	NA

**MELD Score**		0.487	NA
< 10	11/11 (100)		
≥ 10	12/14 (85.7)		

**ALT** (IU/L)		1.000	NA
< 40	4/4 (100)		
≥ 40	19/21 (90.5)		

Data presented as number/total number (percent).

BMI, body mass index; HCV, hepatitis C virus; RNA, ribonucleic acid; APRI, AST-to-platelet ratio index; MELD, model for end-stage liver disease; and ALT, alanine transaminase.

**Table 5:** Treatment adverse events.

**Adverse event**	**Treatment Regimen**		***p*-value**
	**LDV/SOF**	**SOF/VEL**	
Fatigue	26 (29.9)	9 (36.0)	0.561
Insomnia	1 (1.1)	1 (4.0)	0.398
Headache	6 (6.9)	0	0.335
Nausea	5 (5.7)	3 (12.0)	0.374
Abdominal pain	1 (1.1)	0	1.000
Skin rash	7 (8.0)	0	0.346
Arthralgia	3 (3.4)	2 (8.0)	0.310
Thrombocytopenia	6 (6.9)	2 (8.0)	1.000

Data presented as number (percent).

## Data Availability

The Excel sheet data used to support the findings of this study have not been made available because of the hospital policy. Institutional Review Board does not allow the authors to share data with any journal.

## Abbreviations

CHC: Chronic hepatitis C
HCV: Hepatitis C virus
HCC: Hepatocellular cancer
DAAs: Direct-acting antivirals
GT: Genotype
SOF: Sofosbuvir
RBV: Ribavirin
LDV: Ledipasvir
VEL: Velpatasvir
SVR12: Sustained virologic response at 12 weeks posttreatment
APRI: Aspartate aminotransferase-to-platelet ratio index
ESLD: End stage liver disease.
